# Post-Partum Ovarian Vein Thrombosis: Combined Effect of Infection and Factor V Leiden Mutation

**DOI:** 10.4274/tjh.2014.0266

**Published:** 2015-02-15

**Authors:** H. El Farran, A. G. Haddad, A. H. Radwan, A. H. Nassar, R. Hourani, Ali T. Taher

**Affiliations:** 1 American University of Beirut Medical Center, Department of Internal Medicine, Beirut, Lebanon; 2 American University of Beirut Medical Center, Department of Obstetrics and Gynecology, Beirut, Lebanon; 3 American University of Beirut Medical Center, Department of Diagnostic Radiology, Beirut, Lebanon

**Keywords:** Venous thrombosis, Pregnancy, Factor V Leiden, Streptococcal infection

## TO THE EDITOR

Ovarian vein thrombosis (OVT) is a rare complication of pregnancy that mainly affects women in their 3rd or 4th decade. Numerous etiologies have been proposed, including ones of idiopathic origin. Early therapy with anticoagulants can be lifesaving; hence, a high index of suspicion is important in order to avoid serious complications such as pulmonary embolism (14%), sepsis, and death [1]. In this letter, an attempt at uncovering one of the etiologies to further solidify our understanding of the disease is made.

**Figure 1:**
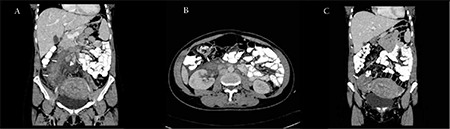
Coronal (A and C) and axial (B) contrast-enhanced multidetector computerized tomography images of the abdomen and pelvis demonstrate an enlarged hypodense right ovarian vein (white arrows, A and B) representing the thrombosed vein, extending from the right adnexa to the inferior vena cava at the level of the right renal hilum. There are severe perivascular inflammatory changes in the retroperitoneum (arrowhead, A). The right ovary is enlarged and heterogeneously enhanced (arrow, C).

A 34-year-old female, gravida 3, para 3, presented on the fifth day after an uncomplicated normal vaginal delivery with right lower quadrant pain, fever, and chills. She had been diagnosed with Behçet’s disease 8 years ago and was maintained on colchicine and steroids for 2 years, which were stopped later. The patient denied previous episodes of deep vein thrombosis (DVT), as well as family history of hypercoagulable diseases. The course of her pregnancy was uneventful except for a positive rectovaginal culture for beta-hemolytic group B streptococci, for which she received prophylactic antibiotics before delivery. She was found to be heterozygous for factor V Leiden. Computed tomography (CT) of the abdomen with IV contrast revealed enlargement of the right adnexa with heterogeneous enhancement and surrounding fat streaking and fluid. It also showed a tubular structure arising from this adnexa extending into the inferior vena cava, where a small filling defect was noted and associated with surrounding fat streaking and fluid (Figure 1). The constellation of findings was suggestive of infection involving the right adnexa with associated thrombophlebitis of the right ovarian vein. The patient was admitted for antibiotics and was discharged on anticoagulation. Repeat CT scan showed gradual resolution of the thrombus. Informed consent was obtained.

The topic of thrombosis in pregnancy has long been the subject of thorough investigation. In western Europe and the United States, maternal thromboembolism is the leading cause of pregnancy-related death [2]. Postpartum, the risk of venous thromboembolism is believed to be increased 20-fold. It is especially significant in the first week after birth [3]. Around one-third of pregnancy-related DVTs occur after delivery [4]. In addition, a link between DVT and infection has been suggested [5]. One of the proposed mechanisms is the alteration of endothelial function in blood vessels after an underlying insult [6,7]. In a recent case series, acute infection in community settings was linked to DVT. The incidence was not found to be related to any specific kind of infection and the conclusion was that acute infection could precipitate DVT [8]. In a systematic review conducted in 2006 about thrombophilia in pregnancy, patients who were heterozygous for factor V Leiden were found to have an odds ratio of 8.32 (CI: 5.44-12.7) [9]. Behçet’s disease should also be kept in mind as a contributing prothrombotic state. It is plausible that this patient’s group B streptococcal infection could have increased her risk of OVT as she had had 2 previous uneventful pregnancies, keeping in mind her increased risk of thrombosis. Further studies to solidify this notion are needed.
